# Staff experiences of a new tool for comprehensive geriatric assessment in primary care (PASTEL): a focus group study: **Primary care staff experiences of geriatric assessment**

**DOI:** 10.1080/02813432.2020.1755786

**Published:** 2020-04-29

**Authors:** Magnus Nord, Carl Johan Östgren, Jan Marcusson, Maria Johansson

**Affiliations:** Department of Health, Medicine and Caring Sciences, Linköping University, Linköping, Sweden

**Keywords:** Care planning, comprehensive geriatric assessment, focus group, frailty, primary care

## Abstract

**Objective:** Comprehensive geriatric assessment (CGA) is recommended for the management of frailty. Little is known about professionals’ experiences of CGA; therefore we wanted to investigate the experiences of staff in primary care using a new CGA tool: the Primary care Assessment Tool for Elderly (PASTEL).

**Design:** Focus group interviews. Manifest qualitative content analysis.

**Setting:** Nine primary health care centres in Sweden that participated in a CGA intervention. These centres represent urban as well as rural areas.

**Subjects:** Nine nurses, five GPs and one pharmacist were divided into three focus groups.

**Main outcome measures:** Participants’ experiences of conducting CGA with PASTEL.

**Results:** The analysis resulted in four main categories. **A valuable tool for selected patients**: The participants considered the assessment tool to be feasible and valuable. They stated that having enough time for the assessment interview was essential but views about the ideal patient for assessment were divided. **Creating conditions for dialogue**: The process of adapting the assessment to the individual and create conditions for dialogue was recognised as important. **Managing in-depth conversations**: In-depth conversations turned out to be an important component of the assessment. Patients were eager to share their stories, but talking about the future or the end of life was demanding. **The winding road of actions and teamwork:** PASTEL was regarded as a good preparation tool for care planning and a means of support for identifying appropriate actions to manage frailty but there were challenges to implement these actions and to obtain good teamwork.

**Conclusion:** The participants reported that PASTEL, a tool for CGA, gave a holistic picture of the older person and was helpful in care planning.

**Key points**
To manage frailty using comprehensive geriatric assessment (CGA) in primary care, there is a need for tools that are efficient, user-friendly and which support patient involvement and teamwork
•This study found that the Primary care Assessment tool for Elderly (PASTEL) is regarded as both valuable and feasible by primary care professionals
•Use of carefully selected items in the tool and allowing enough time for dialogue may enhance patient-centeredness
•The PASTEL tool supports the process of identifying actions to manage frailty in older adults. Teamwork related to the tool and CGA in primary care needs to be further investigated and developed

## Introduction

The growing elderly population is a major challenge for the health care system in developed countries. Although the majority of persons of old age consider themselves healthy and to live an independent life with relatively modest care needs, multimorbidity and frailty increase with age [1,2].

Various scales and instruments to detect and assess frailty have been developed, and many of them have been tested and validated within cohorts [3]. These scales use a wide range of items to capture symptoms or disability measures associated with frailty [4]. Frailty scales provide an estimation of the presence and the degree of frailty, which is valuable for identification, and prognostication of groups or individuals. To manage frailty, other tools are needed. Comprehensive care approaches such as the comprehensive geriatric assessment (CGA) are recommended widely [5]. CGA has been defined as ‘a multidimensional, interdisciplinary diagnostic process focused on determining the medical, psychological, and functional capabilities of a frail elderly person to develop a coordinated and integrated plan for treatment and long-term follow-up’ [6]. There is no consensus about which different items or scales should be included in CGA [7]. The effectiveness of CGA in a hospital setting has been well demonstrated but the evidence is conflicting regarding management in primary care [6,8–13]. According to the British Geriatric Association, CGA adapted to primary care should include ‘a holistic medical review’ resulting in an interactive individualised care plan taking into account personal priorities. This type of intervention is time-consuming; therefore, the selection of individuals for these programmes must be careful [5]. There are a number of instruments and programmes for primary care to support the CGA process, as presented in a recent review, but only a few of them have been tested for validity, reliability and feasibility [14]. They share basic components but are not easily comparable as most of them are presented briefly and the instrument itself is not accessible. Many of them use a set of assessment scales or a large set of items with a risk of being time-consuming and less feasible [15].

Very little is known about professionals’ experiences of CGA instruments in primary care. We found only one study of professionals’ experiences with conducting CGA [16] but the research was not coupled to a specific instrument or to the primary care setting.

In the intervention study, ‘Proactive health care for frail elderly persons,’ a new work model for frail older people in Swedish primary care was tested [17]. Planning this intervention, we found no primary care oriented CGA tool that was suitable for a Swedish primary care context. Therefore, we constructed ‘The Primary care Assessment Tool for the Elderly’ (PASTEL): a four-page form based on the holistic approach of CGA. A tool like PASTEL needs to be feasible to apply and should be regarded as valuable by the users in primary care. Accordingly, we wanted to examine their experiences, which could also add to the scarce knowledge on staff experiences of CGA in primary care in general.

## Objective

The study aim was to investigate the staff experiences of using PASTEL, a tool for Comprehensive Geriatric Assessment in primary care.

## Methods

Three focus group interviews were conducted from November 2017 to March 2018. Focus groups are often used in health research to let individuals with a common experience discuss and share their views and opinions in a way that individual interviews would not permit [18].

### Setting

The new CGA tool (PASTEL) was a part of a proactive intervention for older adults at risk for hospitalisation that was implemented in April 2017 [17]. The intervention took place at nine primary care centres in the county of Östergötland in southeast Sweden. These centres represent urban as well as rural areas and different socioeconomic areas. They have a listed population ranging from 6000 to 21 000 inhabitants. The staff involved in the intervention had no previous experience of geriatric assessment in primary care. The participating primary care centres were presented with a list of patients with increased risk for hospitalisation selected with a statistical prediction model [19]. These patients were invited to an interview with a registered nurse guided by the PASTEL form.

### PASTEL – the CGA tool

Experienced primary care professionals constructed the PASTEL form (Appendix): a general practitioner, a primary care nurse and a physiotherapist. It contains two main parts. The first part is an interview guide for nurses with mostly multiple-choice questions and a self-rating of health that was intended to be performed partly by telephone, followed by a checklist for brief physical examination together with a medication review. It is completed by three open-ended questions to the patient regarding the main concerns about their health and their needs in the future. The second part is used at a team meeting with the responsible physician and the interviewing nurse to make an estimation of frailty with the Clinical Frailty Scale [20] and to decide on the need for further investigations including a checklist of actions to support the older adult.

The intention with PASTEL was:

- To get a broad picture of the health situation of older adults.
- To support encounters where patients could express their own thoughts and wishes.
- To facilitate teamwork between nurses and physicians in primary care.
- To promote actions according to the needs and personal priorities of patients.

### Participants

We gathered a purposive sample with the intention to obtain a high degree of variation. Fifteen participants were invited and three of these cancelled due to a lack of time. The participants were homogeneous in the respect that they all had been part of the project and have used PASTEL. They were heterogeneous in terms of sex, profession, primary care centre (they represented eight out of nine participating primary care centres) and had various levels of experience in the field and different numbers of assessments with PASTEL (Table 1). The participants were invited by e-mail, and written information about the study was included with the e-mail. All participants were also thoroughly informed verbally about the study before the interviews started. The information included the aim of the study, a statement that participation was voluntary and an explanation of how confidentiality was handled. The importance of sharing all experiences about the assessment tool, positive as well as negative, was emphasised.

**Table 1.** List of participants.

Participants	Focus group	Years of work experience	Sex	Number of assessments
Nurse 1	1	8	F	25
Nurse 2	1	11	F	25
Nurse 3	1	11	F	3
GP 1	1	40	M	15
GP 2	1	25	F	5
Pharm 1	2	15	F	35
Nurse 4	2	25	F	3
Nurse 5	2	23	F	8
GP 3	2	25	M	30
Nurse 6	2	33	F	45
Nurse 7	2	34	F	40
Nurse 8	3	38	F	25
Nurse 9	3	10	F	20
GP 4	3	10	M	15
GP 5	3	32	F	8

GP: General Practitioner; F: female; M: male. Nurses were registered nurses or nurse practioners.

**Table 2.** Examples from the analysis.

	Condensed meaning unit	Code	Subcategory	Category
No, so I generally think that this patient group requires more eyes than mine, so to speak.	Patient group requires more eyes.	Teamwork is needed	Challenges for the team	The winding road of actions and teamwork
I tried to focus on his (the patient´s) story and experiences and then she (the wife) supported him when he could not really express what he meant …	Focuses on the patient´s story and the relative supports when needed.	Relatives are supportive	Participation of relatives can be helpful but sometimes unfavourable	Creating conditions for dialogue
That is you get a structured information…yes, it gives a holistic view, right, on all the problems.	Get structured information and a holistic view	Structured information Holistic view	Structure gives the overview needed for action	A valuable tool for selected patients

### Procedures

The focus group interviews took place at three different primary care centres, as this was most convenient for the group members, and lasted for 60–75 min.

The interview guide included three key questions:

1. What are your experiences of using PASTEL?
2. How did you capture the patient’s own expectations of care and thoughts about the future by using PASTEL?
3. What are your experiences of using PASTEL at the team meetings and for care planning?

The last author (MJ) had the role of the moderator and the first author (MN) functioned as a co-moderator during the interviews. The role of the moderator was to promote interaction among the group members and to create an open discussion environment [21]. MN mainly observed the discussion and posed a couple of supplementary questions. Both authors have a broad experience of working with frail patients of old age: MN as a general practitioner and MJ as an occupational therapist in geriatrics. MJ has previous training and experience in qualitative research. MN was responsible for the construction of PASTEL and for the implementation of the tool at the primary health care centres and was thereby already known by almost all the participants.

### Data analysis

The interviews were audio recorded and transcribed verbatim. Qualitative content analysis as described by Graneheim [22] was used to analyse the material. The interviews were analysed in the following steps:

1. Open reading and listening to the interviews to get a good understanding of the content.
2. The meaning units of the text that were important to participants’ experiences of using PASTEL were identified.
3. The meaning units were labelled into codes, resulting in 193 codes.
4. The codes were condensed and sorted into categories and subcategories.

All steps were first carried out separately by authors MN and MJ and then together. The software Open Code 4.03 was used during the analysis. During all steps, a critical discussion was held between the authors to widen their understanding of the content. A third person with extensive experience of qualitative methods and interviews also read the transcribed interviews and served as a discussion partner for each step during the analysis. This person was not involved in the construction of the tool nor the interviews in order to minimise bias in the analysis and enhance the trustworthiness of the study.

## Results

Four main categories and 10 subcategories emerged (Table 3) that describe the participants’ experiences of PASTEL which are presented below.

**Table 3.** Categories and subcategories.

Category	Subcategories
A valuable tool for selected patients	Structure gives the overview needed for action Who is the right patient?
Creating conditions for dialogue	Time and adaptation is important Managing cognitive dysfunction Participation of relatives can be helpful but sometimes unfavourable
Managing in-depth conversations	Specific questions can create deep conversations Talking about the future is valuable but not always appreciated Death – a sensitive subject
The winding road of actions and teamwork	Actions were often initiated during the interview The team-meeting – a starting point for care planning Challenges for the team

### A valuable tool for selected patients

#### Structure gives the overview needed for action

The participants reported that the assessment tool was a valuable instrument in their work with frail older adults. Interviews using PASTEL gave a structured holistic picture of the individual’s health situation and identified the patient’s own thoughts and needs. The participants regarded the interviews as a good preparation for care planning and a means of support to identify actions.

They also stated that PASTEL provided an incentive to take a long-term and preventive approach in their work.

I think for me it has really provided a structure. I think it has been very helpful, and as you say when you talk to your patients and their relatives, this makes you dig a bit deeper, an impression that you almost cover all areas (Nurse 7)

#### Who is the right patient?

Ideas about who was the right patient for assessment with PASTEL differed; some thought the interview could be too extensive and time-consuming for relatively healthy older people. The selection of patients in the intervention resulted in a considerable proportion of non-frail individuals and a great variation in degree of frailty. A few participants considered PASTEL to benefit non-frail elderly people as well. Some pointed out that although they met with well-known patients, the use of PASTEL provided valuable new information. Others found that patients living in nursing homes did not get much benefit from the assessment because their needs had already been identified and met by care planning, but there were examples when valuable information was presented also from these assessments. Many participants stated that it was important to meet the patient when frailty had put the patient in a situation where he/she was in need of action. At the same time, someone argued that health care professionals have to meet the patient before they can really tell if he/she has important needs.

When you say that many patients are really well taken care of (already before the assessment), did you ever think: ‘this was needless… we should have used the resources in another way’ (moderator)

No, I have had a good feeling about it. Still there is a lot that comes to light where we have been able to help in different ways… that can prevent… and I feel that the patients appreciate it … It feels like it gives them security too, a general sense of security. (Nurse 8)

Yes and then to get this helicopter view in a way that we have some difficulties with …even if we have good knowledge we haven´t done the actual evaluation in the way we do now [with PASTEL] (GP4)

### Creating conditions for dialogue

#### Time and adaptation are important

PASTEL interviews lasted about one hour on average. The participants (nurses) who had carried out the interviews described how they tried to adapt the tool to be useful in their work context and to meet the needs of the individual patients. The majority of participants stated that the initial telephone interview of PASTEL was difficult to perform due to, for example, hearing impairment, and that it felt inappropriate to ask some of the questions over the phone. As a result, most participants chose to carry out the entire interview during a visit. This was also believed to be more efficient. The participants reported that they managed to give the patients enough time in a context where they could more easily bring up their own questions compared with a regular medical visit where time often is scarce.

And it gives the opportunity for patients to bring up issues that bother them when they’re at the doctor’s visit, and maybe they feel they want to, but then they notice that the doctor is stressed … And they walk out with their problem anyway, but here they may get the opportunity to bring up something they have thought about for a long time. (Nurse 6)

The majority of the participants preferred to start the interview with practical items like going through the medication list or talking about activities of daily living, saving questions about psychological health and thoughts about the future until later in the conversation. These questions often led to discussions about sorrow and loneliness.

#### Managing cognitive dysfunction

Each patient’s cognitive ability determined how the participants adapted the questions and to what extent they needed to use other sources of information. Some questions needed to be rephrased one or two times to help the patient to express themselves. A few participants found that interviewing patients with more severe cognitive impairment did not add much to the picture, while others found it possible to adjust the questions to obtain valuable information in this context.

I mean that the lady that I am thinking of, she suffers from dementia and has difficulties with answering for herself at all. So I think that an interview will be hard to carry out. (Nurse 3)

For whom should that interview be carried out? (Nurse 2)

You think that you wouldn´t be able to capture what she really wants and thinks about… (Moderator)

No, no exactly! (Nurse 3)

But in the evaluation of that person´s whole situation so to speak then [PASTEL] could be a support, even if you cannot interview her in the same way as if she were cognitively able. (GP 1)

#### Participation of relatives can be helpful but sometimes unfavourable

There was a great deal of interest from relatives in taking part in the interview. In most cases, the relatives provided support for the patient, but there were situations where the nurse took action to meet patients without a relative in order to capture the patients’ own experiences and views.

## Managing in-depth conversations

### Specific questions can create deep conversations

Several participants indicated that PASTEL helped to create in-depth and meaningful conversations. This occurred despite the fact that the majority of the questions were specific, with multiple-choice alternatives. Questions about psychological factors opened up and deepened the conversation. The predominant experience was that the patients were openhearted and had a desire to share their stories. The conversations could be long and could often include stories of grief and loneliness. Sometimes it was difficult to limit the conversation in order to cover all questions during the same visit.

My experience is that, it’s like opening Pandora’s box when they arrive, ‘Someone will listen to me now,’ and for a while I had, like five, six, seven assessment visits when everyone cried… it was like ‘Now it’s my time’ and now everything comes out. It has sometimes been difficult to relate to this because you…there have been other things that needed to be talked about… A lot of grief and loneliness and fear of death and such heavy subjects coming up. (Nurse 1)

### Talking about the future is valuable but not always appreciated

Many of the participants stated that the three open-ended questions about personal priorities and thoughts about future needs in PASTEL were very valuable. However, some of them thought that the questions were somewhat difficult, as the concept of ‘the future’ was considered too imprecise. In addition, some patients were not so interested in reflecting on the future, which gave rise to vague answers.

And when you ask: ‘How do you view your housing situation in the future?’ It is a great question and it is relevant, but many of them, I was really surprised because, I mean they are old and ill, but they do not think a lot about the future. I mean in the way that (laughing): they get along now, they manage, they are OK… So they don’t think about it. (Nurse 7)

Still, the open-ended questions were seen as a good summary of patients’ own thoughts and needs. The answers could include anything from fears about suffering at the end of life to more practical things like how to take care of a wound or get help with cleaning windows.

### Death – a sensitive subject

A couple of participants explained that the subject of death often came up during their interviews while others seldom or never touched on the subject with their patients. A discussion came up in all three focus groups about whether you should formulate specific questions about death and end of life. There were suggestions about having a more direct question about attitudes towards the level of care at end of life to capture fears and worries about suffering, while several participants pointed out that these questions must be personalised and could be inappropriate. Some perceived death as a sensitive subject and had concerns about what reactions could arise if brought up in the wrong context.

## The winding road of actions and teamwork

It became clear in all focus groups that actions to handle frailty to support older people were initiated during the interview, and before and during the team meeting.

### Actions were often initiated during the interview

The majority of the participants found that the interview worked as an action in itself when the nurses were listening, providing information and giving advice. An example of a more long-term intervention that occurred during the interview was the initiation of a process in which the patient became aware of, for example, the need for a change in the housing situation. Actions initiated by nurses before the team meeting included advice on medication management, wound dressing, coordination of home care, contact with occupational therapists regarding technical aids, referral to a physiotherapist or contact concerning activities and social support. Both GPs and nurses agreed that many of these actions would never have taken place without the assessment guided by PASTEL and that these actions could have a preventive effect, for example in preventing falls or making the elderly person more secure in his/her home. Loneliness was perceived as being difficult to deal with, and several participants stated that they had difficulty with finding resources that could provide psychosocial support for older persons in need.

For me it´s quite an important thing about the elderly… many lonely people… they don´t feel well at home and you have to pay attention and listen, or help them to get in contact with someone to talk to, at the church or anywhere…if they manage… (Nurse 4)

However, the process of initiating actions was not uncomplicated. One participant said she thought that discussing, for example home care could raise expectations for patients that were not possible to realise and therefore one should be cautious about proposing different kinds of support. In addition, it was not unusual that patients declined to receive support or other actions. Someone reported that continuity and regular check-ups were what the patients appreciated most.

### The team meeting – a starting point for care planning

The team meeting was primarily a discussion between the nurse who had conducted the interview and the responsible GP, and lasted 10–20 min per patient. Actions at the team meeting could be referral for additional investigation, initiation of home care or change in medication. Participants from the two primary care centres that had a pharmacist in their team appreciated the pharmacist’s participation in the team meeting. The medication review and associated actions were generally regarded as some of the most important parts. Introducing a case manager nurse was an action that was valuable for the majority of the patients according to the participants. The assessment with the Clinical Frailty Scale was a part of PASTEL that was considered important for care planning and provided a moment when members of the team could merge their different views and impressions of the patient’s condition.

### Challenges for the team

There was a consensus among participants that teamwork is essential in the care of frail older adults in primary care. Several referred to the existence of structures in primary care developed for other groups of patients, like dementia or diabetes patients, but which had been previously lacking for this group of patients. They emphasised the importance of having regular team meetings and of limiting the timespan between the interview and the team meeting, as there is a risk of losing more subtle information. Nurses also pointed out the difficulty of presenting information that was said ‘between the lines’ and that was hard to document. There was a lot of responsibility for nurses in the team, which was considered mostly positive as long as there was mutual understanding and a team commitment to the task.

It´s a lot of responsibility on me and that´s fine…but at the same time I've felt that the interest from my doctors to do this together with me has been a bit weak. Especially the team meeting has been a little like: ‘Well we fill in the check-list right like you said here.’ I don´t think that is what´s intended with the team meeting… It should be more precise, like who is responsible and who will do the follow-up (Nurse 9)

Some participants stated that teamwork was facilitated if the GP had good knowledge of and a long-term relationship with the patient. The lack of occupational therapists and physiotherapists connected to the team was considered a problem: these professionals seldom have contact with the primary care centres because they belong to a different organisation, the municipality. However, in small municipalities where there was just one primary care unit, it appears that good collaboration was easier to achieve.

In that respect they are so fortunate in [a small community], you know. Every Thursday morning I meet all the nurses and occupational therapists in the municipality there, so a couple of times when we have had these frail ones… Then I can address it at that meeting so to speak and we decide what to do. (GP1)

## Discussion

### Main findings

The study results suggest that the assessment tool PASTEL assists in performing the important functions of comprehensive geriatric assessment (CGA) in primary care. The participants reported that an assessment with the tool gave a holistic picture of the patient and was helpful in care planning. PASTEL was regarded as beneficial also for pre-frail individuals but was sometimes experienced as too extensive for relatively healthy older adults. Nurses emphasised the importance of having enough time for the interview and adapted the tool to create good conditions for dialogue. These conditions together with patients´ desire to share their stories created deep and significant conversations according to the participants. Actions to support older people were identified and executed both during the interview with nurses and at the subsequent team meeting. PASTEL seems to play a significant role in this process but the participants also described challenges regarding teamwork and carrying out actions.

### Strengths and limitations

A strength with this study is that we formed heterogeneous groups consisting of participants with different professions and levels of experience. In all focus groups, there were participants from two or three primary care centres. The focus group method gave us an opportunity to encourage interaction between the participants. This helped to gather material as rich as possible regarding staff experiences of CGA in our intervention despite that the number of participants was limited.

The interviewing authors have a broad experience of working with frail patients in general practice and geriatrics. The fact that one of the interviewers (MN) was responsible for the implementation of PASTEL might have influenced the participants in their answers making them unwilling to criticise the tool. On the other hand, that may have motivated them to participate and share both positive and negative experiences. At the interviews we therefore let MJ lead the discussion and emphasised that we wanted all kinds of reflections and that their participation could help to improve the tool.

Being part of designing the intervention and working in primary care with frail individuals was an important part of the preunderstanding of the first author (MN). The advantage of this is a deeper understanding of the participants working situation but there is a potential risk of bias in the analysis. To address this issue the authors held critical reflexive discussions during the process of coding and categorising and we triangulated our coding and interpretation with an experienced researcher who was neither part of the intervention nor the construction of PASTEL. Minimising bias was also a reason for us to keep the analysis close to the text and at a low level of interpretation [23].

The experiences of our participants reflect the specific intervention they were part of [17]. They assessed patients selected with a digital prediction model [19], which limits generalisability to some extent. Still, a substantial part of the results reflects experiences of CGA in general, for example the challenge to capture the individual views and preferences of an older adult and to have conversations about the future or end of life. The generalisability of the results is likely to be limited to countries with similar cultures and primary health care systems but some of the findings are probably applicable to a wider context.

### Comparison with existing literature

The study cannot answer the question of how PASTEL performs in comparison with other CGA tools but the participants´ experiences of the value of PASTEL in clinical practice is consistent with descriptions of CGA in previous studies [6,7]. PASTEL also covers most of the areas of unmet needs that older people themselves have identified as important [24]. The challenge of covering all the important aspects of CGA and ensuring that the tool remains feasible in a primary care setting is described by Stijnen et al. in a Dutch setting [25]. In Belgium, GPs perceived that the 300-item MDS-HC (Minimal Data Set-Home Care) was too extensive and that it gave little added value in establishing a personal management plan [15]. We did not use multiple validated scales for different aspects of frailty in PASTEL, but focused more on creating dialogue based on a relatively low number of questions. In addition to checking various items, the nurse must establish a trusting relationship with the elderly person to get a full picture of the patient’s needs and to ensure acceptance of care [26]. A prerequisite for this is having enough time and letting patients share their stories [27]. Even if most of the nurses in our focus groups were experienced in communication, they still thought that PASTEL helped them to create in-depth conversations. The fact that PASTEL has an open structure with room for adaptation may have contributed to this. In a previous focus group study on staff experiences from both hospital and community care in Sweden, participants shared the view that experience and competence was more important than standardised testing [16]. This is in line with the views of our participants though they didn´t have previous experience of CGA. To adapt the assessment to the individual to focus on indicators of quality of life and the person’s own values, rather than using a lot of scales, was considered a high priority also in the recommendations from the American Geriatrics Society Expert Panel on Person-centred Care [28].

The challenge of tackling loneliness among elderly people was stressed by our participants. Evolving strategies to deal with this is essential. In a recent complex intervention study from England, a combination of different social activities and strengthening of networks in the local community showed promising results [29].

The selection of patients for this kind of thorough assessment is difficult, but is important in order to achieve meaningful actions, which is also stressed in frailty guidelines and in earlier studies [5,30]. The patients in this study were selected using a digital method and were invited to participate as a part of the intervention. A noticeable proportion of the selected patients was rather healthy or pre-frail older adults, which probably explains why the participants in some cases found the assessment too extensive. At the same time, someone stated that often you have to meet the individual to be able to assess frailty in a reliable way. In the absence of a perfect screening method for frailty, we believe that an assessment tool like PASTEL should support adaptation to various stages of frailty. The interviewer should be able to conduct a shorter interview when there are no apparent signs of frailty and to prioritise more time for individuals with greater needs.

Continuity of care has been proven important, especially for patients with multimorbidity [31–33]. The value of a long-term doctor-patient relationship was brought up several times in the interviews [34]. For the more frail individuals with insufficient autonomy, there is an additional need for a nurse to take the role of a case manager [35]. This function forms a bridge between patients and their doctors and helps to coordinate care for the patients.

The teams at the primary care centres consisting of a nurse and a physician have probably an effective size for most occasions, but there is a need to expand the team in more complicated cases. This was pointed out as a concern by both GPs and nurses and is a demanding task for the integrative care system. The role of PASTEL and the function and organisation of a team for CGA need to be further investigated, considering that they are an essential part of CGA, and earlier research has indicated that CGA is less effective when it is performed by individual professionals compared with a team [36,37].

### Meaning of the study

To provide good health care for the increasing population of frail elderly persons, primary care must evolve proactive strategies for recognition and treatment. Structured assessment is the first step in creating individualised care plans and there is a need for assessment tools that are both feasible and efficient in the primary care setting. We conclude that the CGA tool PASTEL was perceived as feasible and helpful in care planning. Furthermore, the tool can be useful for patients’ involvement in care planning and thus supports a person-centred care approach in CGA.
